# 4539 Building a Translational Science pipeline: The Indiana CTSI STEM K-12 Program

**DOI:** 10.1017/cts.2020.203

**Published:** 2020-07-29

**Authors:** Elmer Sanders, Vanessa Barth, Leigh-Ann Cruz, Ilesha Sherrer, Jacob Olson, Emily Speidell, Elvia Solis, Sharon Harrison, Amy Hinshaw, James A. McAteer

**Affiliations:** 1 Indiana University School of Medicine; 2 Eli Lilly and Company; 3 Cardinal Ritter High School; 4 Pike High School; 5 Decatur Central High School; 6 Decatur Central HS; 7 Arsenal Technical High School; 8 Indiana CTSI K-12 STEM Program; 9 McKenzie Center for Innovation and Technology; 10 IU School of Medicine

## Abstract

OBJECTIVES/GOALS:

- Develop strong network of science teachers interested in promoting scientific research to their students.
- Place students in an immersive summer research internship that, when possible, matches their career interests.
- Expose students to the numerous career paths within the STEM field.

METHODS/STUDY POPULATION:

- The program recruits socio-economically disadvantaged students and provides them a stipend, and also accepts students who can participate unpaid.
- Local school teachers are engaged in a summer fellowship to learn biotechnologies and research. In Spring these teachers help recruit students and during the subsequent Fall help students with college and scholarship applications.
- Students are placed in a variety of laboratories within the Schools of Medicine, Science, Dentistry, Public Health, Informatics, Health and Human Sciences, Engineering and Technology, especially in biomedical engineering. Students are also placed in industry laboratories such as Eli Lilly and the Indiana Bioscience Research Institute.
- Long-term program follow-up is done through post-internship surveys to assess impact on graduate and professional school admission.

RESULTS/ANTICIPATED RESULTS:

- Since the Indiana CTSI was established in 2008, 872 students have participated in the summer internship.
- 71% of past interns are underrepresented minorities in science or classified as disadvantaged by NIH criteria.
- 17% of students interned during grade 10, 72% during grade 11, and 11% during grade 12.
- 21% of students engage in the program for more than one year.
- 100% of past interns are currently enrolled in or have graduated college.
- Over 60% of those with a bachelors degree proceed to graduate and professional schools and over 80% stay in STEM related fields. These rates are equal for interns from underrepresented minorities or those classified as disadvantaged by NIH criteria.

DISCUSSION/SIGNIFICANCE OF IMPACT:

- Students engaged in the Indiana CTSI STEM program are progressing through the translational science pipeline based on their graduating from college and remaining in the STEM field.

